# Usp7 protects genomic stability by regulating Bub3

**DOI:** 10.18632/oncotarget.1989

**Published:** 2014-05-19

**Authors:** Serena Giovinazzi, Pietro Sirleto, Vasilisa Aksenova, Viacheslav M. Morozov, Roberto Zori, William C. Reinhold, Alexander M. Ishov

**Affiliations:** ^1^ Department of Anatomy and Cell Biology, University of Florida College of Medicine, Gainesville, FL; ^2^ University of Florida Health Cancer Center, Gainesville, FL; ^3^ Bambino Gesu' Children's Hospital, Rome, Italy; ^4^ Molecular Pharmacology laboratory, Institute of Technology and Institute of Cytology, St-Petersburg, Russia; ^5^ Department of Pediatrics, University of Florida College of Medicine, Gainesville, FL; ^6^ Genomics and Bioinformatics Group, Laboratory of Molecular Pharmacology, National Cancer Institute, National Institutes of Health, Bethesda, MD

**Keywords:** deubiquitinase USP7, Bub3, spindle assembly checkpoint (SAC), genomic instability, USP7 inhibitors

## Abstract

USP7 (Ubiquitin Specific processing Protease-7) is a deubiquitinase which, over the past decade emerged as a critical regulator of cellular processes. Deregulation of USP7 activity has been linked to cancer, making USP7 inhibition an appealing anti-cancer strategy. The identification of novel USP7 substrates and additional USP7-dependent cellular activities will broaden our knowledge towards potential clinical application of USP7 inhibitors. Results presented in this study uncover a novel and pivotal function of USP7 in the maintenance of genomic stability. Upon USP7 depletion we observed prolonged mitosis and mitotic abnormalities including micronuclei accumulation, lagging chromosomes and karyotype instability. Inhibition of USP7 with small molecule inhibitors stabilizes cyclin B and causes mitotic abnormalities. Our results suggest that these USP7-dependent effects are mediated by decreased levels of spindle assembly checkpoint (SAC) component Bub3, which we characterized as an interacting partner and substrate of USP7. *In silico* analysis across the NCI-60 panels of cell lines supports our results where lower levels of USP7 strongly correlate with genomic instability. In conclusion, we identified a novel role of USP7 as regulator of the SAC component Bub3 and genomic stability.

## INTRODUCTION

As it was first postulated by the German biologist Theodor Boveri in 1902, the precise partitioning of duplicated chromosomes to daughter cells is essential for the development and survival of all organisms. Defects in segregation lead to aneuploidy, the state where chromosomes are gained or lost. Several lines of evidence indicate that aneuploidy contributes to the evolution of cancer [1-5] and genomic instability [6], a hallmark of tumor cells [7].

During mitosis, at the prometa/metaphase transition, many sister chromatids do not achieve the necessary bipolar orientation [8]. Abnormal attachments (syntelic or merotelic) must be corrected so that until all chromosomes are under proper tension and bi-orientation, the metaphase-anaphase transition is delayed [9, 10]. In eukaryotic cells this delicate mission is assigned to the Spindle Assembly Checkpoint (SAC) [11]. To the SAC belongs a long list of proteins [12] that survey, detect and correct failed or improper microtubule/kinetochore attachments. This system prevents segregation errors by inhibiting APC/C^Cdc20^ mediated destruction of cyclin B and securin, thereby blocking anaphase entrance. The SAC is satisfied once all mis-attachments are corrected, than Cdc20 binds and activates APC/C to ubiquitinate cyclin B and securin for proteolytic degradation [11, 13]. Drop in securin levels lift the inhibition on the cysteine protease separase. This enzyme then cleaves the cohesin rings which hold sister chromatids together, to initiate metaphase to anaphase transition [14].

Thus, maintenance of a functional SAC is fundamental for genome stability. Genetic studies on hypomorphic or heterozygous mice for the components of the SAC, demonstrated that these mice are characterized by elevated aneuploidy [15-17] and increased rates of tumors: spontaneous for Mad2+/- [17], CENP-E+/- [18, 19] and Bub1 hypomorphic [20, 21]) or carcinogen-induced for Bub3 [15] and BubR1 [16].

The protein Bub3 (budding uninhibited by benzimidazoles 3 homolog) [22] is a key SAC component that is associated with unattached kinetochores [23]. Here it recruits other components of the Mitotic Checkpoint Complex (MCC), such as Bub1, to suppress the APC/C function by either sequestering or inhibiting Cdc20 [24, 25]. In addition, Bub3 is necessary to establish the formation of correct microtubules-kinetochore attachments [26, 27]. Cells with reduced levels of Bub3 compromise localization of the other components of the SAC, Bub1 and BubR1 [27], failing SAC activation [26]. Due to the inability to sense and correct kinetochores-microtubules attachments cells with reduced expression of Bub3 display chromosome segregation problems in the form of micronuclei [28], misaligned or lagging chromosomes [27].

The deubiquitylating enzyme USP7 is a critical regulator of cellular processes through regulation of stability of tumor suppressors, such as p53 and PTEN [29] [30, 31], DNA damage response proteins [32], transcription factors [33, 34], viral proteins [35-39], epigenetic modulators [40-44] and mitotic regulators [45, 46].

Here, we found that USP7 depletion causes genomic instability characterized by abnormal chromosomes segregation, accumulation of micronuclei and increased aneuploidy. In addition, reduced levels of USP7 strongly correlate with genomic instability across the NCI-60 cell line platform. We demonstrated that these mitotic abnormalities in USP7 depleted cells are mediated by SAC protein Bub3. USP7 interacts with Bub3 in mitosis; this binding favors Bub3 stabilization as cells depleted by USP7 have reduced levels of Bub3. These effects are occurring in similar extents in p53 wild type (wt) and null cell lines.

The results presented in this study provide the first evidence that loss of USP7 leads to genomic instability by destabilizing one of the key mitotic checkpoint components.

## RESULTS

### USP7 Depletion Causes Genomic Instability

To better understand pleotropic functions of USP7 in controlling cellular processes, we decided to generate USP7 shRNA cells on p53+ and p53- background. In our previous study [46], while generating USP7 shRNA cells, we noticed several interphase nuclei abnormalities that are usually recognized as derived from mitotic segregation defects [47]. Cells with reduced levels of USP7 have high levels of nuclear blebs and micronuclei (MN) compared to control-depleted cells (Fig. S1); we observed this phenomenon in both p53 wt HEp2 and p53 null H1299 cells. In USP7 shRNA cells, a small sub-population of cells positive for USP7 does not have nuclei abnormalities (such as ill shaped nuclei, massive micronucleation, nuclear buds and nucleoplasmic bridges), suggesting that they are caused by USP7 depletion (Fig. S1).

### Depletion of USP7 leads to increase in micronuclei formation

To study the significance of these nuclear abnormalities, we decided to quantify the micronuclei (MN). MN formation is the manifestation of chromosomal instability and a consequence of mitotic abnormalities [48]. MN originate from chromosomal fragments or whole chromosomes that are not properly attached to spindle microtubules [49]. At the end of mitosis, nuclear envelope reforms around the ‘lost’ genetic material in the cytoplasm, where MN appear as tiny nuclei by DNA staining [50].

**Fig. 1:**
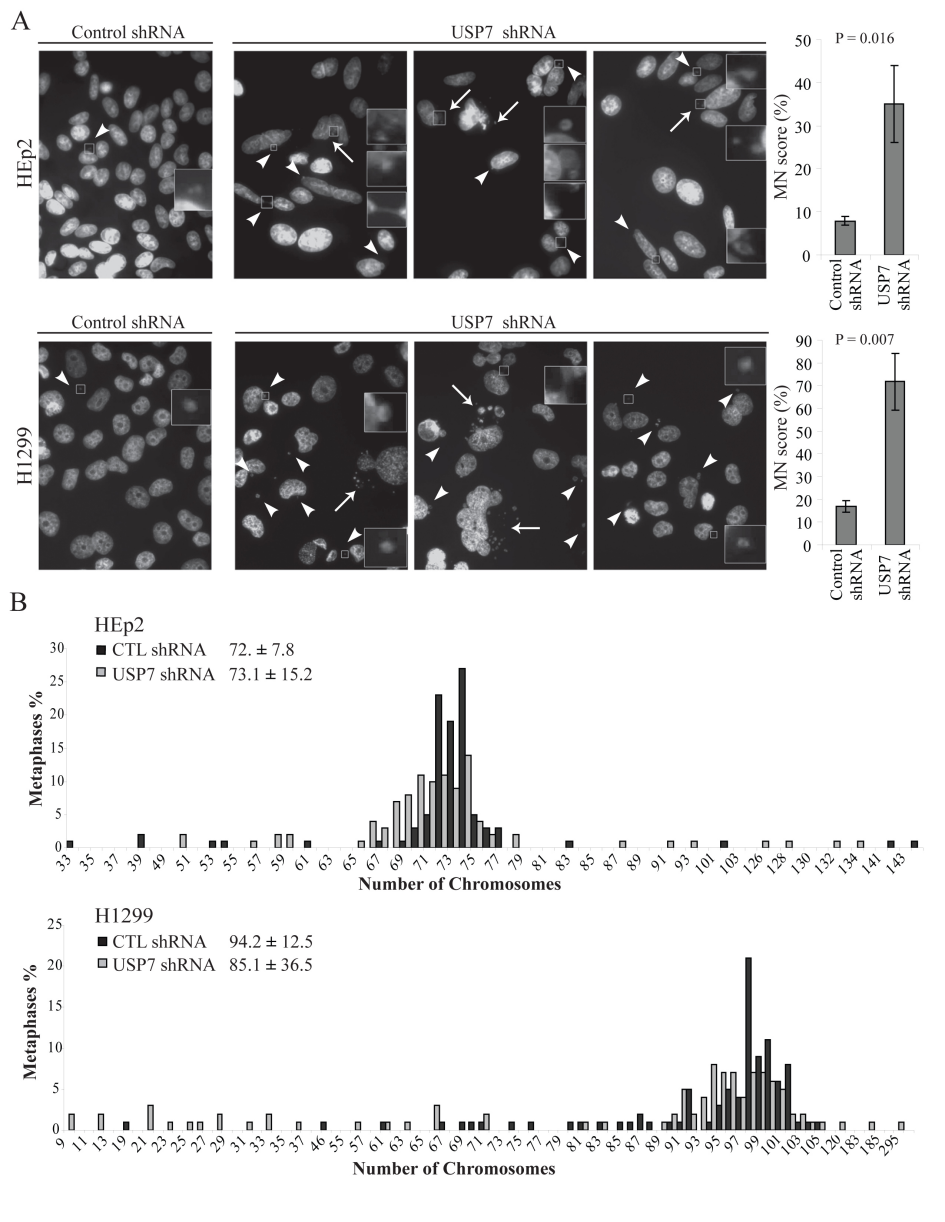
Depletion of USP7 increases genomic instability (A) Micronuclei (MN) analysis in HEp2 and H1299 cells. Representative fields show MN in control and USP7 shRNA cell lines. MN are heterogeneous in both size and number of MN *per* cell: white arrowheads indicate single MN while white arrows point to groups of MN. Right: quantifications of MN accumulation over the total number of cells (MN score). For each experiment a minimum of 300 cells were counted (± SD, n=3) and scoring MN according to the criteria previously described [51]. (B) Karyotype analysis of HEp2 and H1299 cells stably depleted by control or USP7 shRNAs. Results were generated from 100 metaphases. Numbers represent modal chromosome number (sample's weighted average ± weighted s.d.).

Depletion of USP7 elevated MN formation in both p53 positive HEp2 and p53 negative H1299 cells (Fig. 1A, left for representative images). Only occasional MN were detected in HEp2 and H1299 control depleted cell lines (Fig. 1A: left, first panel in enlarged box). To statistically assess the accumulation of MN upon USP7 depletion, we adopted the previously established rules for MN scoring [51]. A dramatic increase of MN score was observed in USP7 depleted cell lines (Fig. 1A, right).

In mammalian cells, MN form from lagging chromosome fragments – in case of failed repair of DNA double strand breaks [48], or whole chromosomes – upon 1) defects in assembly of kinetochore [52, 53] and non-functional SAC [12, 47, 54, 55], or 2) spindles abnormalities [56, 57]. We found that the MN population was heterogeneous in USP7 shRNA cells in regards of both size and number *per* cell, as shown by the pictures in three distinct fields (Fig.1A). We could clearly distinguish two different MN sub-populations. The first one was characterized by multiple and larger MN (Fig.1A, arrows). This type of MN usually arises from multipolar mitoses due to the inability of the cell to correctly partition groups of chromosomes. The increased incidence of multipolar events upon USP7 depletion and the underlying mechanism were previously published by our group [46]; thus, multiple and large MN can originate from the multipolarity induced by Aurora A accumulation. The second MN sub-population was represented by a small MN, often located between two interphase nuclei (Fig. 1A, arrowheads). Since this type of MN derives from lagging chromosome at the anaphase onset [58], it is likely that depletion of USP7, in addition to multipolarity, could induce other mitotic segregation problems. Collectively these data indicates that USP7 depletion may cause genomic instability, one of the hallmarks of cancer [7].

### Depletion of USP7 elevates aneuploidy

To understand in detail the role of USP7 down-regulation in genomic instability, we next analyzed the karyotypes of HEp2 and H1299 cells stably expressing control or USP7 shRNAs. Our expectation was to observe deviation from the cell line modal chromosome number (calculated as an average number of chromosomes *per* mitotic plate for 100 cells).

Karyotypes of the analyzed cell lines (Fig. 1B) were nearly triploid for HEp2 cells with modal chromosome number of 72 ± 7.8, and nearly tetraploid for H1299 cells with chromosomal modal number of 94.2 ± 12.5. However, upon USP7 depletion, a deviation from these numbers was observed, that is a characteristic of aneuploidy[59]. In p53 positive HEp2 cell line, the calculated chromosomal modal number was 73.1 ± 15.2. The doubling of the standard deviation indicates an increased aneuploidy in these cells. More dramatic effects were observed in p53 negative H1299 cell line, in which the modal chromosome number was reduced from 94.2 to 85.1 chromosomes. As well, the standard deviation of modal chromosome number was increased almost three-fold. Consistently with the appearance of MN (Fig. S1 and 1A), the number of cells with gain or loss of chromosomes was increased in USP7 shRNA cells in both p53 positive and negative cells (Fig. 1B), with a general tendency in reduction of chromosomes number.

### USP7 Interacts with SAC Protein Bub3 and Controls its Stability

**Fig. 2:**
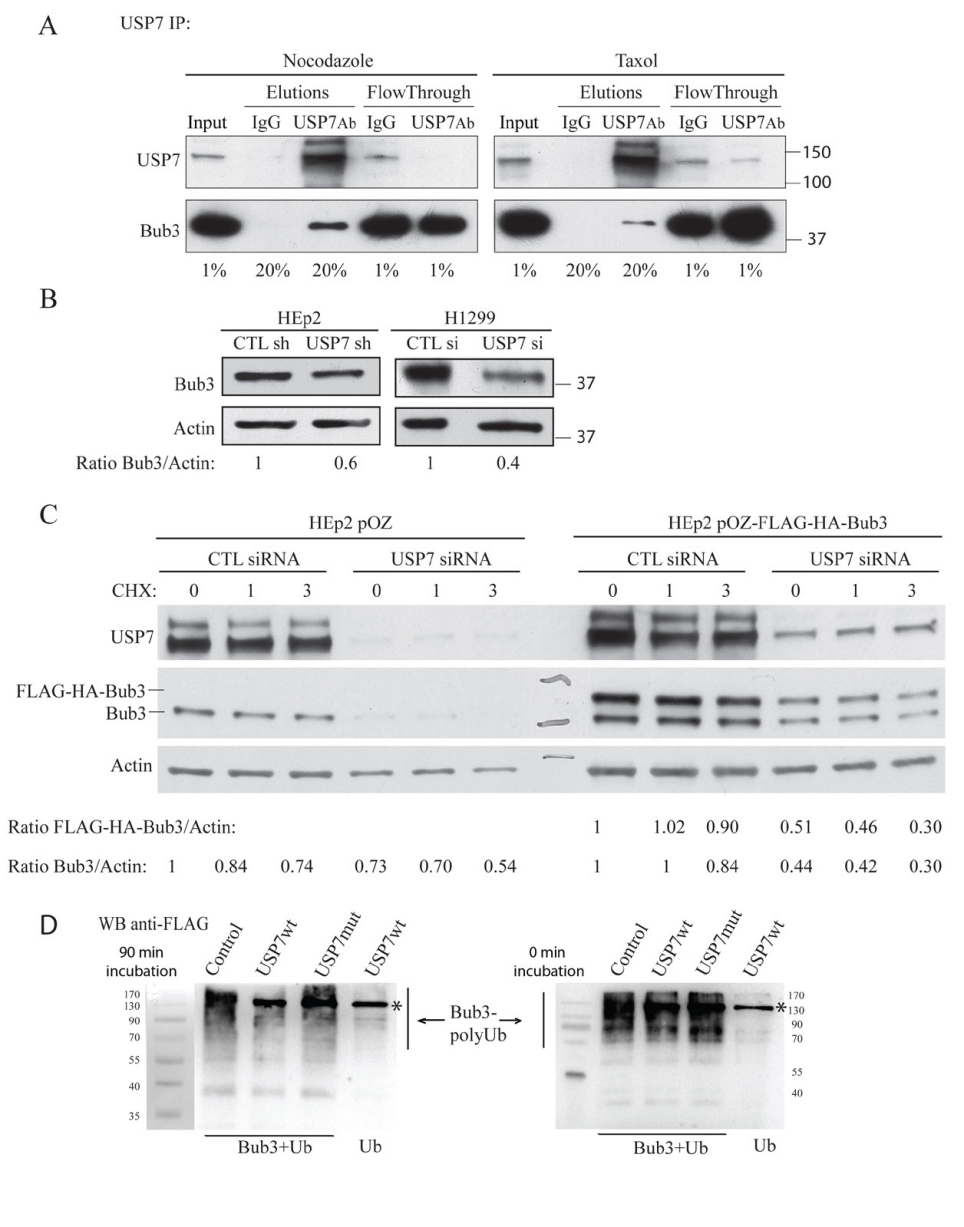
USP7 interacts with Bub3 and regulates its stability (A) Immunoprecipitation experiments of endogenous USP7 in HEp2 cells synchronized in mitosis by nocodazole or Taxol exposure. Bub3 is pulled down only with USP7 antibody but not with IgG control. Numbers below the blots represent the sample percentile loaded. (B) Analysis of Bub3 protein levels in control- and USP7 depleted HEp2 and H1299 cells. Numbers below the blot represent relative quantification of Bub3 signal over actin chosen as loading control. (C) Analysis of protein levels and stability of Bub3. HEp2 cells expressing Bub3 (HEp2 pOZ-Bub3); control: empty vector (HEp2 pOZ) transfected with control (CTL) or USP7 siRNA and treated with cycloheximide (CHX) for 0 (control), 1 and 3 hours. Numbers below the blot represent relative quantification of Bub3 signal over actin. (D) *In vitro* analysis of Bub3 deubiquitination by USP7. Poly-ubiqutinated Bub3 were purified from НЕК293Т cells co-transfected with HA-Bub3 and FLAG-Ub (Bub3+Ub). Purified proteins were treated with lysates purified from HEK293T cells: non-transfected (Control), wild type (FLAG-USP7wt) and catalytically inactive USP7 (FLAG-USP7mut) for 90 min (left) and 0 min (right). Lysates from cells transfected with FLAG-Ub alone (Ub) and treated with USP7wt were used as a negative control. Samples were analyzed with anti-FLAG antibodies that recognize poly-Ub Bub3 and USP7-FLAG (marked with *). Note disappearance of poly-Ub Bub3 in sample treated for 90 min with USP7wt, but not with USP7 mutant or control lysates. Data show a representative experiment out of three.

We observed that USP7 depletion induced genomic instability that may result from a change in stability of mitotic checkpoint proteins. While we were screening for mitotic proteins which would have differential stability upon USP7 depletion, the ‘interactome’ landscape of human DUBs was published [60]. This report indicated that USP7, among other proteins, interacts with SAC protein Bub3 in HeLa cells. To test USP7/Bub3 interaction, immuno-precipitation experiments of endogenous USP7 were done in HEp2 cells synchronized in mitosis by either nocodazole or Taxol exposure (Fig. 2A). In both conditions, Bub3 was pulled-down by USP7 specific antibodies indicating that endogenous proteins, Bub3 and USP7, interact *in vivo*.

Next, we sought to investigate Bub3 protein stability in USP7 depleted cells. Bub3 protein levels were decreased upon USP7 depletion, in both HEp2 and H1299 cells (Fig. 2B). To confirm these results we generated HEp2 cell lines over-expressing FLAG-HA tagged Bub3. USP7 depletion caused a decrease in Bub3 protein levels and stability of Bub3 (cycloheximide treated cells, CHX), for both endogenous and over-expressed protein (Fig. 2C).

Data presented on Fig. 2C suggested that Bub3 is a new substrate for USP7 deubiquitinating activity. To answer this directly, we performed *in vitro* deubiquitination assay on poly-Ub Bub3 using USP7 wt and USP7 catalytically inactive mutant [36]. Incubation of poly-Ub Bub3 with USP7 resulted in disappearance of poly-Ub Bub3 bands, while incubation with USP7 mutant did not affect poly-Ub species (Fig. 2D). Thus, Bub3 not only interacts with USP7, but is a new substrate of this DUB.

**Fig. 3:**
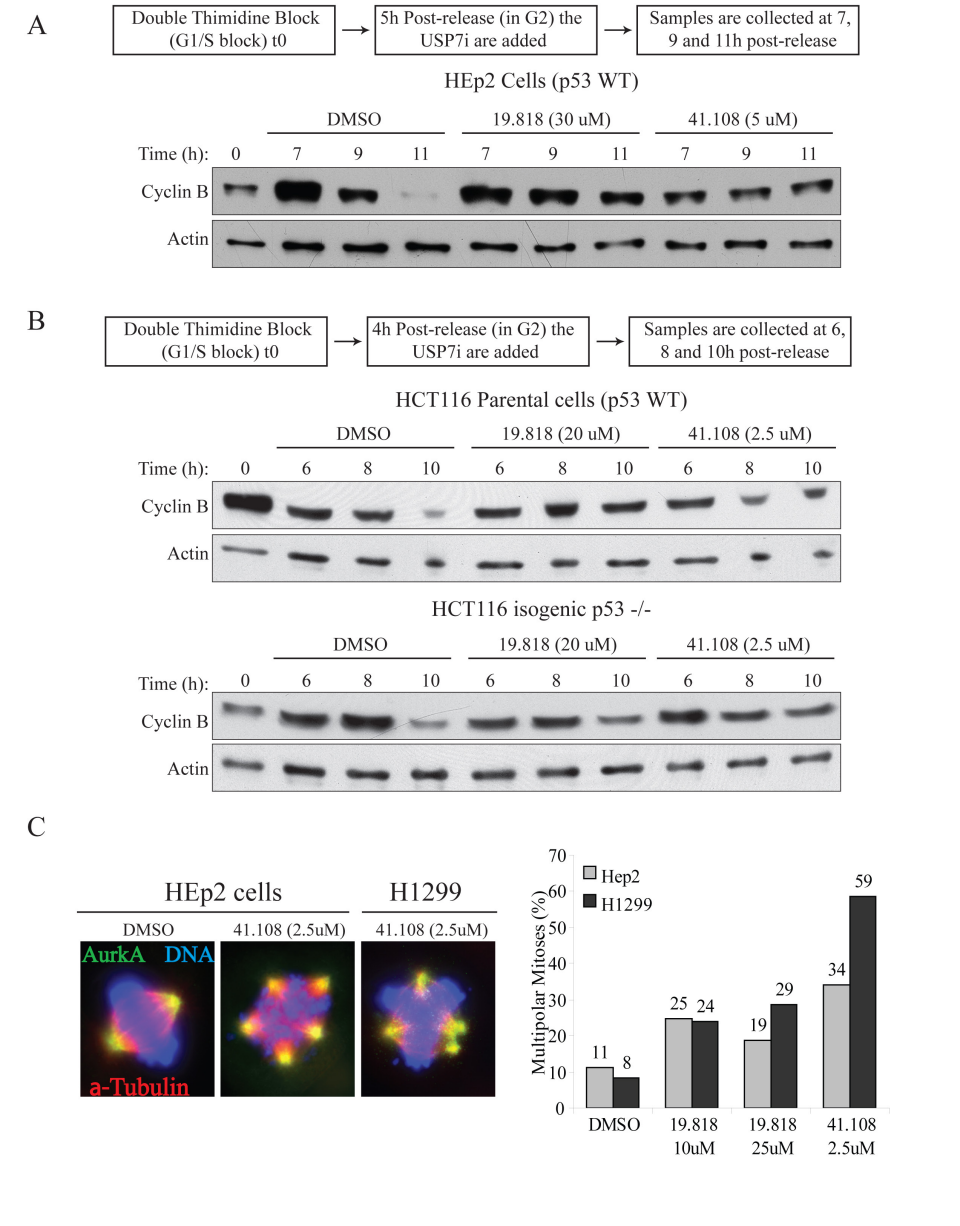
Inhibition of USP7 stabilizes cyclin B and induces multipolarity (A) HEp2 (p53+) cells were synchronized by DTB (S-phase block, 0h); USP7 inhibitors (USP7i), HBX 19.818 [61] and 41.108 [62] or DMSO (vehicle) were added 5 hours after DTB release (scheme on top); samples were collected at 7, 9 and 11 hours after DTB release. (B) HCT116 parental (p53+) and isogenic p53-/- cells were synchronized by DTB (S-phase block, 0h); USP7i were added 4 hours after DTB release (scheme on top); samples were collected at 6, 8 and 10 hours after DTB release. In all cell lines, USP7i protects cyclin B stability. (C) USP7i cause AurkA protein stabilization (not shown) which leads to accumulation of multipolar events in both cell lines. Left, IF for AurkA (green), α-tubulin (red), DNA (blue); right, quantification of multipolar events; >200 mitoses *per* sample.

**Fig. 4:**
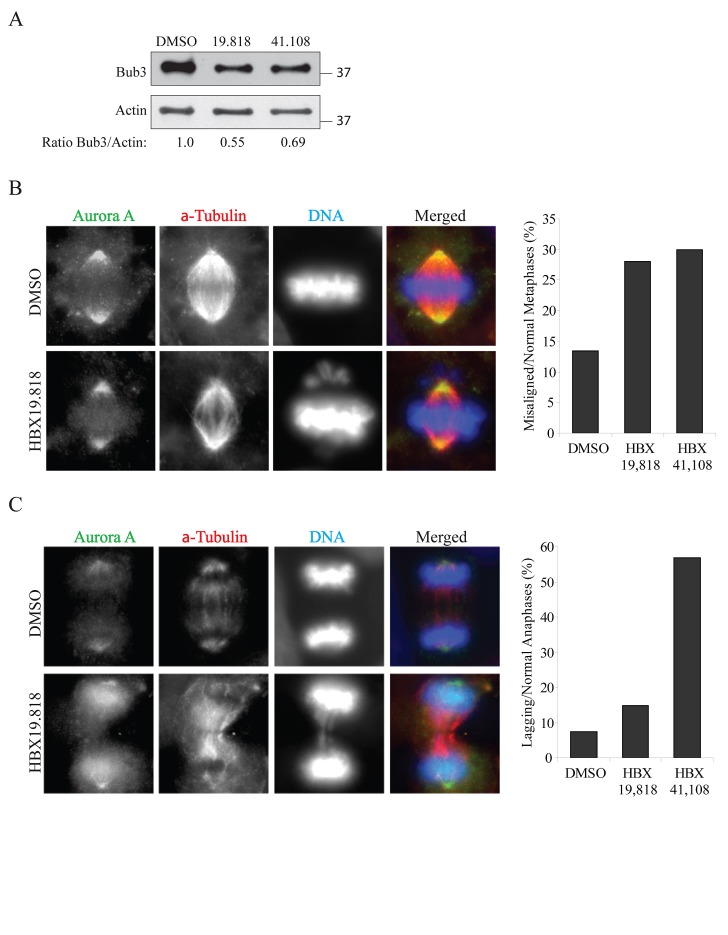
Inhibition of USP7 causes mitotic abnormalities (A) Analysis of Bub3 protein levels in HEp2 cells treated as described in Figure 3. The samples loaded are the 11 hour time-points from DTB release (6 hours of USP7 inhibition). Numbers below the blot represent relative quantification of Bub3 signal over actin chosen as loading control. Levels of Bub3 are decreased upon USP7 ihibition. (B) Left: Representative images for the immunofluorescence staining of HEp2 cells treated with 10 μM of HBX 19.818 for 24 hours. Right: quantifications of lagging chromosomes over the total number of anaphases in HEp2 cells treated for 24 hours with DMSO, 10 μM HBX 19.818 or 2.5 μM HBX 41.108. For each experiment a minimum of 100 anaphases were counted. (C) Left: Representative images for the immunofluorescence staining of HEp2 cells treated with 10 μM of HBX 19.818 for 24 hours. Right: quantifications of misaligned metaphases over the total number of metaphases in HEp2 cells treated for 24 hours with DMSO, 10 μM HBX 19.818 or 2.5 μM HBX 41.108. For each experiment a minimum of 200 metaphases were counted.

To exclude off-target effects from si/shRNAs, we next tested two USP7 inhibitors on Bub3 stability. First we assessed whether these inhibitor recapitulated the published effects of USP7 depletion on cyclin B and Aurora A stability [46]. Both inhibitors HBX 19.818 [61] and HBX 41.108 [62] stabilized cyclin B in both p53 wild type cells (HEp2 and HCT116 parental, Fig. 3A and B) and p53 null (HCT116 p53-/-, Fig. 3B, and H1299, not shown). In addition, cells exposure to these drugs caused Aurora A protein accumulation (not shown) with consequent increase of multipolar mitoses (Fig. 3C, representative images left, multipolar mitoses calculation right). Since these inhibitors reproduced the data from USP7 depletion [46] we monitored Bub3 stability upon USP7 inhibition in HEp2 cells. Both HBX 19.818 and HBX 41.108 caused decrease in Bub3 protein levels (Fig. 4A). These results further suggest that Bub3 is a direct target of deubiquitinating activity of USP7.

### USP7 depletion increases misaligned metaphases and lagging chromosomes

**Fig. 5:**
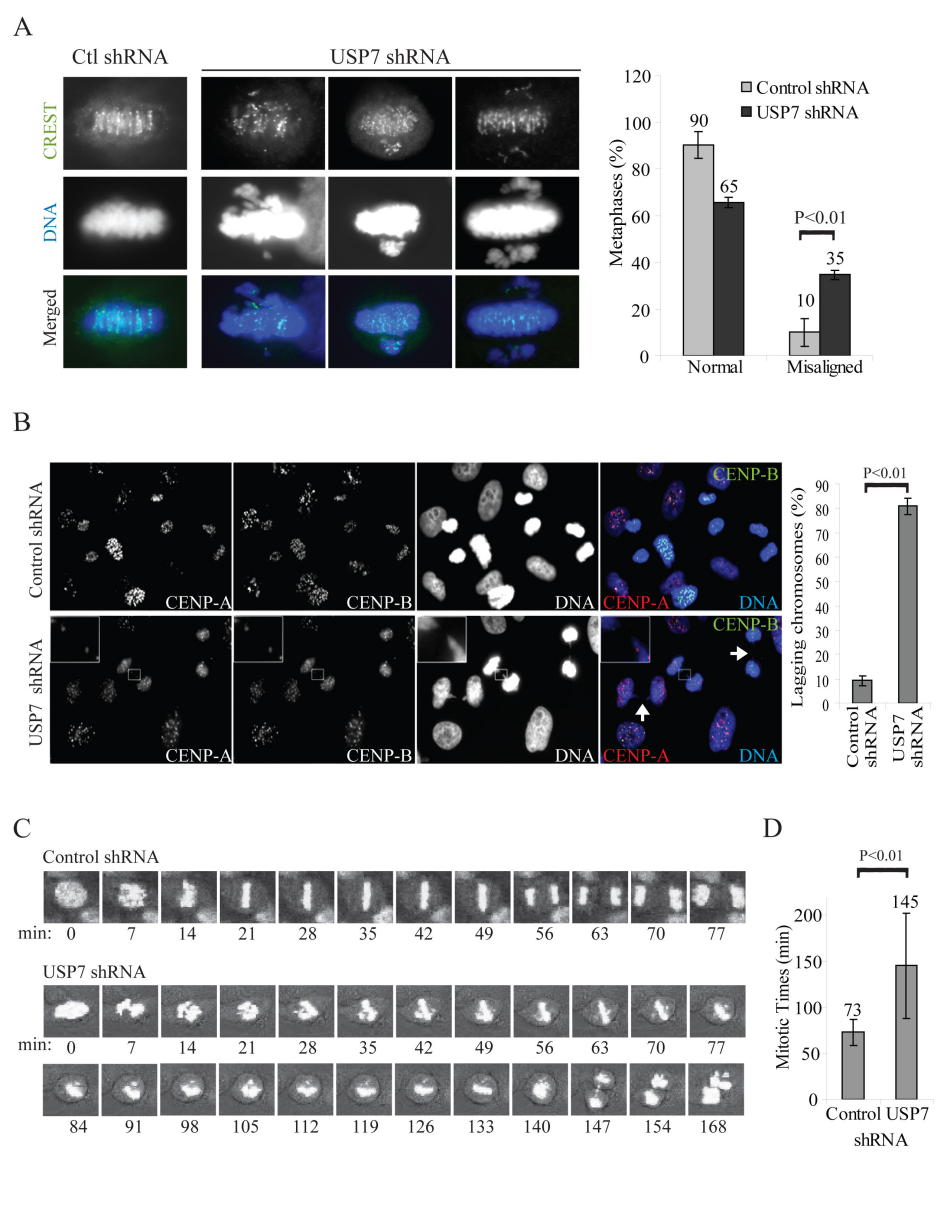
USP7 depletion causes mitotic abnormalities (A) Immunofluorescence staining of HEp2 cells stably expressing control or USP7 shRNAs, labeled with centromere autoantibodies (CREST, green) and DNA (blue); three different metaphases are shown. Right panel: quantification of misaligned metaphases in HEp2 cells control and USP7 depleted. Misaligned metaphases with chromosome pulled from multiple poles were excluded from this analysis. A total of 440 metaphases were counted over three independent experiments. (B) Left: immunofluorescence staining of HEp2 cells labeled with centromeric proteins CENP-A (red), CENP-B (green); DNA: blue. Inset: representative lagging chromosome; arrows: bridges. Right: quantifications of lagging chromosomes over the total number of anaphases (lagging chromosomes %). For each experiment a minimum of 300 anaphases were counted (± SD, n=3). (C) Time lapse snapshots of HEp2 control or USP7 depleted cells, using the NEBD as time 0. (D) Average mitotic timings in HEp2 control or USP7 depleted cells monitored by time lapse microscopy from the NEBD to cytodieresis (± SD, n=13).

One of the mechanisms responsible for the MN formation is the presence of chromosomes-spindle attachments defects [58]. In USP7 depleted cells we observed a substantial increase of metaphases with misaligned chromosomes (Fig. 5A), as it was observed upon Bub3 depletion [26]. Similarly, anaphases with lagging chromosomes were detected in almost all mitotic events in HEp2 cells stably depleted of USP7 (80% of lagging chromosomes *versus* 10% of control shRNA cells, p=0.00002; Fig. 5B). We confirmed these data exposing cells to USP7 inhibitors, scoring for misaligned metaphases and lagging chromosome exclusively in cells with bipolar spindles (Fig. 4B and C).

Altogether, these data strongly indicate that USP7 depletion causes significant chromosome mis-segregation that may be driven 1) by multipolarity due to accumulation of Aurora A [46] and 2) from misaligned and lagging chromosomes. To discriminate between the two scenarios, we performed time lapse microscopy on HEp2 control and USP7 shRNA cells transiently expressing histone H2b-GFP. Control depleted cells progressed normally through mitosis with an average time of 73 minutes from nuclear envelope breakdown (NEBD) to cytodieresis (Fig. 5C, D). However mitoses in cells depleted by USP7 were prolonged, to an average of 145 minutes (Fig. 3C, D), with several unaligned and then lagging chromosomes that eventually formed MN in the daughter cell cytosol (Fig. 5C bottom, see frames 147-168).

These results are consistent with the increase of MN, genomic instability (Figs. 1 and S1) and the data previously published for Bub3 [12]. Indeed many reports demonstrated that Bub3 localizes to unattached kinetochores to recruit and interact with other members of the MCC to maintain Cdc20 inactive until all kinetochores are correctly attached and aligned to the mitotic plate [23, 26-28]; moreover, haplo-insufficiency of Bub3 leads to chromosomal mis-segregation and chromosomal instability [28, 63].

### USP7 gene expression compared to genomic instability (NCI-60)

**Fig. 6:**
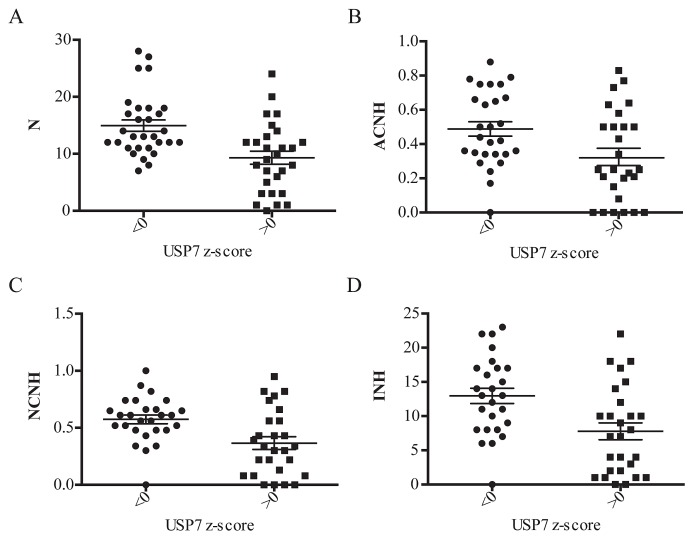
Correlation analysis of USP7 transcripts levels and genomic instability in NCI-60 cell line panel The transcript z-score on the x-axis was graphed against parameters of genomic instability on the y-axis; scatter plots ± SEM. In each case the genomic instability data is split into those with cell lines with USP7 transcript values less than or greater than 0, that is cell lines above or below the 60 cell line mean. (A) USP7 expression versus numerical complexity (N); p-value=0.0003. (B) USP7 expression versus the fraction of abnormal chromosomes that that experience numerical heterogeneity (ACHN); p-value=0.0084. (C) USP7 expression versus the fraction of normal chromosomes that that experience numerical heterogeneity (NCNH); p-value=0.0016. (D) USP7 expression versus the index of numerical heterogeneity (INH); p-value=0.0015.

We demonstrated that depletion of USP7 leads to genomic instability. To further address this correlation, we next analyzed USP7 expression across the NCI-60 cell line panel relative to several parameters of genomic instability [64], as 1) numerical complexity (N), 2) fraction of abnormal chromosomes that experience numerical heterogeneity (ACHN), 3) fraction of normal chromosomes that experience numerical heterogeneity (NCNH), and 4) the index of numerical heterogeneity (INH) (Fig. 6). For all chosen parameters, we observed correlation of increased levels of genomic instability and reduction in USP7 mRNA levels, confirming results of USP7 experimental depletion described earlier in this paper.

Prior genetic and RNAi studies demonstrated that decreased Bub3 weakens the spindle checkpoint machinery and impairs cells from correcting ill-formed attachments between microtubules and kinetochores without accelerating mitosis [13, 27]. These findings may explain the observed mitotic abnormalities upon USP7 silencing and inhibition. Hence, we can conclude that cells with reduced levels of USP7 present aneuploidy and genomic instability due to decreased levels of Bub3 protein (Fig. 1, 2, 4 and 5); this correlation was also reproduced across the NCI-60 cell lines panel (Fig. 6).

## DISCUSSION

In this study, we identified a new biological role of USP7 in G2/M progression and characterized Bub3 as a USP7 interaction partner and a USP7 substrate. We presented evidence for a novel, p53-independent, role of USP7 in maintenance of genomic stability, thus suggesting a tumor suppressor function for this protein. Results presented in our previous work [46] provided evidence of a new USP7 function in mitosis and in response to the anti-mitotic drug Taxol. The current study indicates that the loss of USP7 is linked to elevated genomic instability, another factor that undermines taxane cytotoxicity and treatment [65, 66]. This work indicates that USP7 is essential in maintaining genomic stability in mammalian cells, independently from the cell tissue of origin and p53 status. Indeed analysis of interphase cells with reduced expression of USP7 presented several manifestations of mitotic abnormalities such as ill shaped nuclei, massive micronucleation, nuclear buds and nucleoplasmic bridges (Fig. S1 and 1A). These events are generally considered hallmarks of tumor cells due to the genomic instability of the cancerous growth.

Closer analysis of mitotic figures revealed that loss of USP7 causes a wide array of mitotic abnormalities such as multipolar spindles[46], increase of MN formation (Fig. S1 and 1A), accumulation of misaligned metaphases and lagging chromosomes (Fig. 5). Karyotype analysis of HEp2 and H1299 cells stably expressing control or USP7 shRNAs revealed that USP7 depleted cells were more aneuploid than the control depleted cells, presenting changes in the modal chromosome number (Fig. 1B); moreover, we recapitulated the same results upon transient depletion of USP7 in non-tumorigenic MCF10A breast epithelial cells (not shown). Collectively these data indicates that USP7 depletion causes genomic instability, one of the key hallmarks of cancer [7]. Interestingly, a previous report analyzing USP7 interacting landscape identified one of the key SAC components, protein Bub3, as a potential USP7 interacting partner [60]. Our results clearly demonstrated that endogenous USP7 and Bub3 interact in mitosis (Fig. 2A).

Next, we tested whether the loss of USP7 would cause Bub3 destabilization. A marked reduction of Bub3 upon USP7 depletion in H1299 and HEp2 cells was observed (Fig. 2B), affecting both accumulation and stability of endogenous and over-expressed protein (Fig. 2C), suggesting that Bub3 is a novel target of deubiquitinating activity of USP7. Unfortunately, our attempts to isolate ubiquitinated forms of Bub3 in presence of MG132 in control or USP7 depleted cells failed (not shown). However, *in vitro* experiments (Fig. 2D) confirmed that Bub3 is a direct substrate of USP7 DUB activity.

Prior genetic and RNAi studies demonstrated that decreased Bub3 causes inactivation of the spindle checkpoint machinery thus ablating the ability to correct ill-formed attachments between microtubules and kinetochores. These findings may explain the observed mitotic abnormalities upon USP7 silencing. Hence, we can conclude that cells with reduced levels of USP7 are impaired in the spindle checkpoint assembly and signaling due to decreased levels of Bub3 protein, resulting, finally, in elevated genomic instability (Fig. 1 and 5); this correlation was also reproduced across NCI-60 cell lines panel (Fig. 6).

In regard of mitotic timing, it was shown that Bub3 depleted HeLa cells presented disorganized anaphases but the times between NEBD and anaphase were unaffected [13]. In our experiments we observed that mitotic timing was considerably longer by cyclin B stability or time lapse microscopy in USP7 depleted cells ([46], Fig. 5C and D) and in cells exposed to USP7 inhibitors (Fig. 3A and B). Consistently with our published results, depletion or inhibition of USP7 causes prolongation of mitotic timings (Fig. 5C, [46]), which cannot be attributable exclusively to Bub3 reduction. It will be interesting in future studies to evaluate USP7 participation in mitosis through regulation of additional substrates. Intriguingly, a recent report showed that another deubiquitinating enzyme, USP44, is relevant to mitotic progression. USP44 is thought to act as checkpoint by deubiquitinating Cdc20 [67]. In addition, it was recently shown that USP44 prevents chromosome aneuploidy independently from its mitotic checkpoint role. USP44–/– MEFs displayed an increased in lagging chromosomes in anaphase which seem to arise from abnormal or incomplete centrosome separation at NEBD [68]. The mechanism by which USP44 would regulate genome stability is not fully understood yet, but both USP44 DUB activity and its ability to bind centrin appear to be indispensable for this function [68]. Since some of the effects of the loss of USP44 and USP7 are similar, it will be interesting in the future to assess whether these two DUB enzymes cooperate in ensuring mitotic progression and genome stability.

In conclusion, we identified a new biological role of USP7 in G2/M progression and characterized Bub3 as a new USP7 interaction partner and potential substrate. We presented evidence for a novel, p53- independent, role of USP7 in maintenance of genomic stability, thus suggesting a tumor suppressor function for this protein. Results presented in our previous work [46] provided evidence of a new USP7 function in mitosis and in response to the anti-mitotic drug Taxol. The current study indicates that the loss of USP7 is linked to elevated genomic instability, which could be another factor that decreases efficacy of taxane cytotoxicity and treatment [65, 66]. In addition, our study offers insights on USP7 as a therapeutic target. Small molecule inhibitors of USP7 DUB activity were developed to activate G1/S block that supposes to trigger p53 mediated cell death [69, 70]. Efficacy and employment of these drugs is limited by mutations or inactivation in p53 gene, occurring in >50% of human cancers, including breast cancer [71] [72]. Our previous study [46] warned for future clinical use of these USP7 inhibitors in combinatorial regimens with taxanes, as depletion of USP7 increases taxanes resistance. However, considering the genomic instability derived from USP7 depletion, the current study may suggest that a combination of USP7 inhibitors with DNA damaging agents may represent a successful strategy in killing tumor cells.

## METHODS

### Cell Culture and Drugs treatments

HEp2, H1299, HCT116 parental (p53+) and isogenic p53-/- cells were cultured in Dulbecco's modified Eagle's medium (DMEM) supplemented with 10% fetal bovine serum, 2 mM glutamine and 100 U/mL penicillin and 100 μg/mL streptomycin (Gibco BRL, Carlsbad, CA) and grown in a humidified 5% CO2 incubator. Concentrations of USP7 inhibitors HBX 19.818 [61] and HBX 41.108 [62] are indicated in the corresponding figure legends.

HEp2 cells stably expressing Bub3 were created using the TAP system pOZ-FH-N [73].

### Transient and Stable Depletions

For transient siRNA transfections (Dharmacon, Thermo Fisher Scientific, Waltham, MA) were used according manufacturer instructions. Control shRNA and shRNA targeting USP7 were previously described [46, 74].

### Immuno-precipitation Analysis

Cells synchronized by addition of 10nM Taxol or 10 uM Nocodazole for 16 hours were lysed for 15 min at RT in lysis buffer consisting of 50 mM Tris-HCL (pH 7.45), 150 mM NaCl, 1mM EDTA, 1% Triton X-100, in the presence of 10 mM N-ethylmaleimide (Sigma, St. Louis, MO), 5 mM iodoactetamide (Sigma), 1 mM phenylmethylsulfonylfluoride (Calbiochem, EMD Chemicals, Gibbstown, NJ), 1 mg/mL aprotinin (Sigma), 1 mM leupeptin (Sigma), 1 mM pepstatin (Sigma). Lysate was then pre-cleared by centrifugation at 1800g for 10 min at RT, and filtration trough 0.45 micron filter (Corning). Pre-cleared lysates (Input) were incubated with normal rabbit antibodies (Santa Cruz) or USP7 antibody (Bethyl labs, Montgomery, TX) conjugated to Protein G magnetic beads (Dynabeads, Invitrogen-Life Technologies, Grand Island, NY) for 2 hours at RT. Beads were thoroughly washed and eluted with 0.1 M Glycine pH 3.

### Deubiquitination Assays

НЕК293Т cells were transfected with pcDNA-Bub3-HA, pFLAG-USP7wt, catalytically inactive USP7 (pFLAG-USP7mut [36]) and pcDNA3-FLAG-Ub. 24 hours after transfection, HEK293T cells were treated with MG132 (1 μM, ApexBio) for 16 hours. Immunoprecipitation of FLAG-targeted proteins was done with anti-Flag M2 Affinity Gel (Sigma) according to manufacturer's protocol. Proteins were eluted with 3X FLAG peptide in 100 μl of TBS (50 mM Tris HCl pH 7.4, 150 mM NaCl, protein inhibitors). For *in vitro* deubiquitination, Bub3-Ub and Ub precipitates were incubated with equal amount of USP7 wild type and USP7mut enzymes. Reactions were incubated for 90 min in deubiqutination buffer (20 mM Tris, 50 mM NaCl, 0.1% Triton X-100) at 30° C and stopped by adding 5X Laemmli Loading Buffer. Samples were separated by SDS-PAAG, and probed with anti-FLAG antibody (Sigma).

### Antibodies for Western Blotting

Primary antibodies to USP7 rabbit (Bethyl labs, Montgomery, TX), HA (Covance, Princeton, NJ), Bub3 (BD Transduction Laboratories, San Jose, CA), and Actin (Sigma) were diluted in 3% milk/PBS-Tween 0.05% and incubated overnight at 4°C. Densitometry analysis was performed using the Quantity One software (Bio-Rad, Hercules, CA).

### Immunofluoresence

Immunofluoresence analysis was completed as previously described [74]. In brief, cells were fixed, permeabilized and incubated with the following primary antibodies: USP7 (Bethyl labs), CENP-A (Abcam, Cambridge, MA), CENP-B (Santa Cruz, Santa Cruz, CA.) or CREST (human autoimmune antibodies recognizing several centromere proteins) following by incubation with corresponding secondary antibodies. Images were analyzed using a Leica TCS SP5 confocal microscope.

### Microscopy Analysis

Micronuclei (MN) scoring: cells stably depleted by control or USP7 shRNAs and grown on coverslips were fixed and stained with Hoechst for DNA analysis according to immunofluorescence procedure. The criteria adopted for MN scoring were previously described [51, 75]. MN score represent the number of MN *per* cell. For each sample at least 300 cells were counted from three experiments.

For the quantification of misaligned chromosomes we adopted previously described procedure [76]; a chromosome was considered mis-aligned if it localized in an area outside of the 40% of the mitotic spindle. In this analysis, misaligned chromosomes resulting from the presence of multiple poles were not scored. At least 100 metaphases were scored for each experiment (SD±3).

Time-lapse imaging of cells was performed as described before [77]. Briefly, HEp2 control or USP7 shRNA cell lines were transiently transfected with GFP-histone H2B (gift from Dr. Duane Compton, Dartmouth); cells were treated as described and analyzed by Leica TCS SP5 confocal microscope equipped with environmental chamber; images were taken every 7 min.

### Metaphase Spreads and Karyotyping

HEp2 and H1299 cells stably expressing control or USP7 shRNAs were treated with 50 ng/mL colcemid (Invitrogen-Life Technologies) for 2 hours before proceeding with metaphases preparations. Cells were collected and resuspended in a hypotonic solution of 2% KCl and 2% Na3C6H5O7 for 7 minutes at 37°C. Metaphase spreads were then prepared and stained with Giemsa-trypsin (G-band) procedure. Analysis was carried out using the OLYMPUS BX41 microscope equipped with a BASLER scA1400-17gmASI digital camera. Images were analyzed using the Applied Spectral Imaging (ASI) software V7.0.6.8860. Unless stated differently, for each experiment at least one-hundred metaphases for each sample were counted.

### NCI-60 gene transcript expression quantification

Gene transcript expression was derived from the integration of probes from five microarray platforms. These include the Affymetrix (Affymetrix Inc., Sunnyvale, CA) Human Genome U95 Set (HG-U95, GEO accession GSE5949); Human Genome U133 (HG-U133, GEO accession GSE5720), Human Genome U133 Plus 2.0 Arrays (HG-U133 Plus 2.0, GEO accession GSE32474); and the GeneChip Human Exon 1.0 ST array (GH Exon 1.0 ST, GEO accession GSE29682), and the Agilent (Agilent Technologies, Inc., Santa Clara, CA) Whole Human Genome Oligo Microarray (WHG, GEO accession GSE29288) [78].

Normalization using GCRMA for the Affymetrix, and GeneSpring GX for the Agilent were done as described previously [78]. Quality control based on a minimum intensity range of < 1.2 log2, probe number (a minimum of 2 and a maximum of 122), and probe versus probe correlations within genes were done as described previously [78]. Probe intensity transformation to average z-scores were determined for each gene for each cell line as described previously [78].

### Genomic instability parameters

The parameters numerical complexity (N), the fraction of abnormal chromosomes that that experience numerical heterogeneity (ACHN), the fraction of normal chromosomes that that experience numerical heterogeneity (NCNH), and the index of numerical heterogeneity (INH) are described and presented in [64].

## SUPPLEMENTARY FIGURE


