# Supplementary figures and images for: Usp7 protects genomic stability by regulating Bub3
Source: Oncotarget. 2014 May 19;5(11):3728–42. doi: 10.18632/oncotarget.1989 (PMC4116516; doi:10.18632/oncotarget.1989)

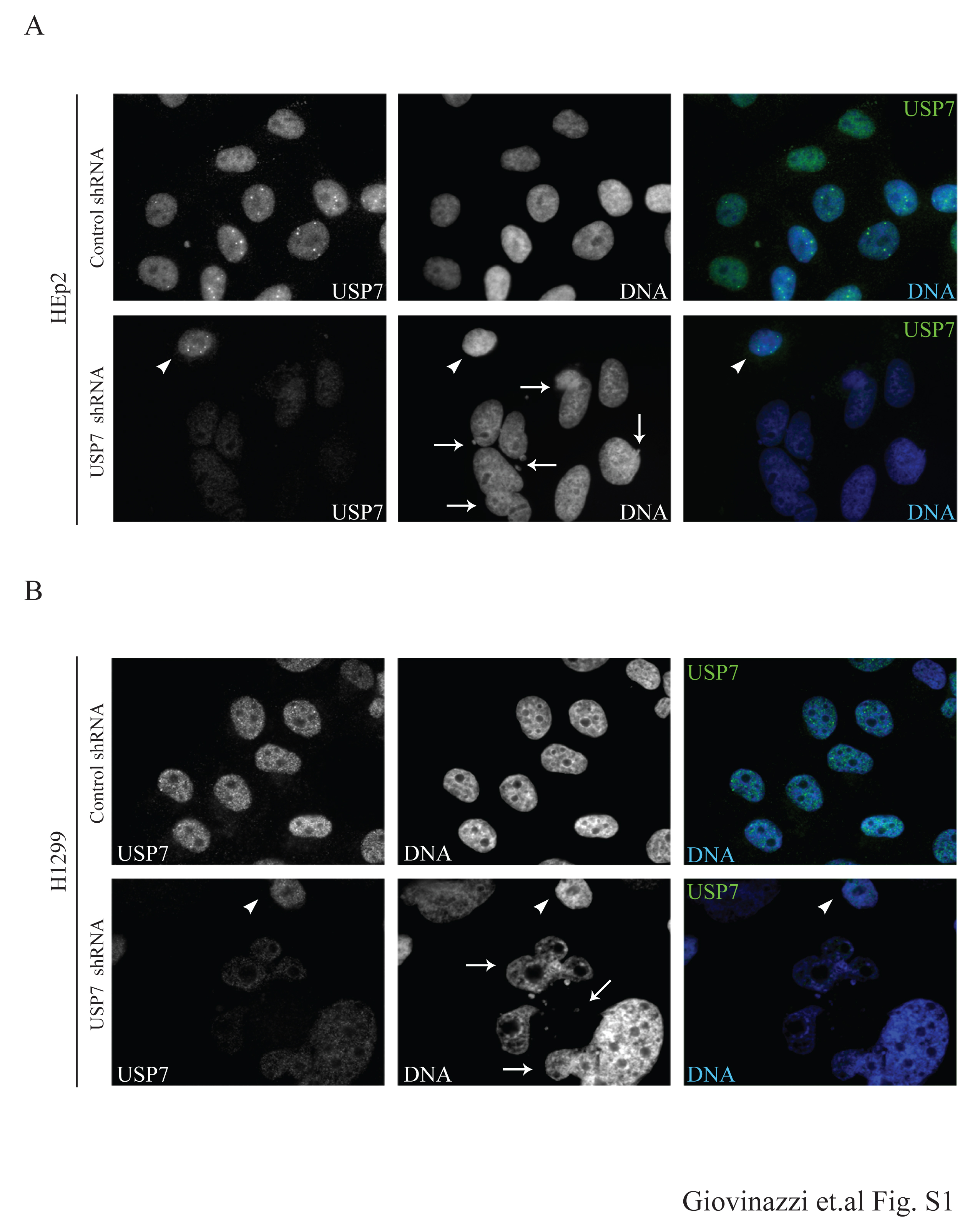

Supplement: Supplementary file 1 [file oncotarget-05-3728-s001.jpg]
